# A Remarkable Case of Poisoning with Lead, Extending over a Period of Nearly Four Years

**Published:** 1850-12

**Authors:** 


*A Remarkable case of Poisoning with Lead, extending over a period of nearly four years.* By Edward Murphy, M. D., of New Harmony, Indiana.

Mr. R——, merchant, aged 42, of medium height, and rather stout habit of body; of bilious temperament, and sound intellect; has always enjoyed good health, and no hereditary liability to disease; has always been temperate, and a close but active business man. During September, 1843, had a slight attack of autumnal fever of short continuance, and throughout the following winter had been often afflicted with pains in his abdomen, which disturbed him a good deal.

About the last of February, 1844, was confined to his bed for several days, with excessive, intermitting abdominal pain, and obstinate constipation of his bowels; but, he thinks, without fever; and was treated by his physicians for an attack of acute peritonitis. The constipation was very obstinate, and only yielded after several days, to very large doses of medicine. But, I consider it impossible, that acute inflammation within the abdomen should have continued so long as this attack did, without producing some organic change among the abdominal viscera.

After an imperfect recovery, Mr. R. went to Louisville on business, during the following March, where he was again attacked with the same symptoms—though not quite of the same severity—and was attended by a distinguished physician, who pronounced his disease to be abdominal neuralgia, stating, that it was a rather frequent complaint among mercantile men in that place, and prescribed accordingly. He also gave it as his opinion, that his former attack was the same disease and not peritonitis. Since that time, Mr. R.'s complaint has been considered neuralgia, and treated as such.

From that time up to the 22d of February, 1846, he has been suffering almost constantly, with excessive pain in his abdomen, radiating from thence to all parts of his body, often of very great severity; obstinate constipation of his bowels, accompanied often with nausea and vomiting, (the patient attributing the nausea and vomiting to the *very* large doses of opium which he was sometimes obliged to take;) was frequently confined to his bed; he lost flesh and strength, notwithstanding a constent good appetite, and had a bloated though anæmic countenance. He had very much the appearance of a person in cachexia from malignant disease. There was a dirty yellow color of the skin, and a yellow discoloration of the albuginea oculi simulating jaundice, the whole time. Sometimes during this period, he became affected with slight paralysis of the extensor muscles of the fingers of the right hand, with the exception of the index, which rendered him unable to write; his vision became imperfect; there was great mental prostration, approaching hypochondriasis,—indeed, he was totally unable to do business, throughout the greater part of this period, from mental imbecility, sometimes being unable to perform the minutest calculation, or to attend his customers, who generally considered him insane; was very irritable the whole time

About this time, Mr. R. was attacked with what was thought to be apoplectic fits, having had four or five, and on the 24th, I was called in consultation. He was confined to his bed, very pale and feeble; sensible, although very week in mind; would give an answer, in relation to his case, and immediately forget that he had done so; sometimes became alarmed at persons present, and again was much terrified at absent imaginary enemies, who were conspiring against him—a state resembling delirium tremens; speech, faltering and hesitating; sight, defective. His face was frequently affected with choreic convulsions, when he would complain of severe shooting pains through his body, and of which he was in constant dread; tongue, soft and broad; pulse, feeble, but almost natural as respects frequency; bowels, constipated; stomach, very irritable; chest, perfectly sound; sounds and rhythm of the heart natural; nothing unusual in the appearance of his urine; although very feeble, would sit up for a short time when desired. Considering it impossible that an individual should have had four or five fits of apoplexy in two or three days, without any lesion to the brain or symptoms denoting such, and on carefully interrogating his family—the physician in attendance not having seen him in a fit—I made out his attacks to be of an epileptiform character—being preceded by the horrid scream of epileptics, accompanied with evident convulsions. I advised opening the bowels by active purgatives, opiates, nourishing diet, blister to the nuchæ, and sulph. quinia, when the bowels were well opened, and took my leave, after assuring his family that I did not consider his present attack to be apoplexy, but probably a part of his old complaint, and gave an unfavorable prognosis.

Mr. R. remained in nearly the same state, but without another fit, until the 3d of March, when I was again called in and associated in the treatment of his case. By persistence in the above remedies, to which was added wine and brandy, he very gradually recovered to his late state of health. When so far recovered as to be able to sit up, his defective vision became almost complete amaurosis, which continued some time, then gradually disappeared, but was not entirely recovered from; the patient was fully of the opinion, that it was caused by the quinia he had been taking, although never more than six grs. in twenty-four hours, and with no idiosyncrasy as to its action. There was also at this time increased paralysis of the right hand, the left also becoming slightly paralysed.

From the 16th of March, at which time my attendance ceased, up to January, 1847, when he placed himself in my hands for treatment of fistula of the anus, complicated with fissure, he continued to have the same attacks, of greater or less severity, with only short intervals of repose, being nearly worn out with constant suffering and bad health. As opium was his only relief, he generally prescribed for himself throughout the lengthened period of his sickness, except when his attack was unusually severe. After the cure of his fistula, his disease returned with greater severity, and of a more alarming appearance than ever.

On the 10th of June, in the absence of his regular physician, I was again consulted. Mr. R. was confined to his chamber and almost to his bed, the mere wreck of his former self. Scarcely able to sit up, weeping from excruciating pain, and in such a state of mind as to express a wish to commit suicide, and indeed he was afraid he should do so. His face, pale and wan, was marked by the deepest despair, from extreme suffering, imploring me strongly for relief; wrists entirely dropped from complete paralysis—being perfectly helpless, and unable to straighten either hand, unless by the aid of the opposite arm, and requiring all the care of an infant, in being fed, washed, &c., yet, a comparatively good gripe with his hands. His extremities were dwindled away to the mere sheaths of the muscles; his abdomen seemed to be the centre as usual, from which his pain radiated, and it was with the greatest difficulty that I could persuade him, after a careful examination, of the non-existance of organic disease there. The slightest touch of the skin over the umbilicus, and indeed over other parts of the body, produced such terrific pain as almost to throw him into convulsions, producing all the effects of an electric shock; while the greatest presure over the same place, gave him no uneasiness, but rather relief: his bowels were always constipated, unless moved by medicine—was the constipation produced by the large quantity of opium which was taken, or did it depend on paralysis of the muscular tunic of the intestines? there was sometimes vomiting of a greenish watery fluid; tongue, flat and broad; pulse, very feeble, and more frequent than natural; his cachetic appearance was that of a person in the last stage of malignant disease; apetite, comparatively good; his suffering was much more intense during the night than the day, unless relieved by excessively large doses of opium. From the balls of both thumbs, which were much atrophied, execruciating pains would arise, shooting with great severity up his arms and shoulders, to the back of his neck and head; the shoulders were affected with constant pain, especially the deltoid muscles, which also were slightly paralyzed. The pain in his lower extremities was also very severe, commencing in the soles of his feet, which were so sore that he dreaded to touch the floor with them, and shooting up the limbs to the lumbar region with dreadful suffering. There was also at this time a new source of suffering, shooting pain through his testicles, of such severity, as almost to produce fainting; indeed, to see him in his suffering, was the most heart-rendering sight I ever witnessed, and I was greatly astonished to see how any human being could so long survive so much and such constant misery.

I stated to Mr. R., which I had done several times before, though not when attending him, that he presented in the strongest light, all the symptoms of poisoning with lead, and had it been possible that he could in any manner have been exposed to its influence, I should have no hesitation in attributing all his suffering and bad health to that cause. But, Mr. R. was a merchant, and in no way liable to be acted upon by lead or any of its salts in his business. There were no lead pipes or utensils used about the house; nor, had he taken it in any form as medicine during his whole life. The autumn before the commencement of his sickness, he built a new store and repaired his house, which were painted in the usual manner; and this was the only exposure to the influence of lead to which he could refer. I however considered, that this could not be the cause in itself, as I thought it impossible that its influence could have extended over a period of nearly four years.

His case seemed perfectly hopeless, and I firmly believed he would never leave his chamber again alive. As *all* the remedies recommended for neuralgia had been exhausted without any benefit, and as he had taken so much medicine from time to time, that his stomach gave way almost at the bare mention of it, I felt very much at a loss what to devise. I, however, advised Mr. R. to submit to an alterative course of mercury as a last resort; giving him to understand that I considered neuralgia, convulsions, and various anomalous affections, might depend upon a cachectic state of the body, from poison either taken into, or generated within it, and preventing its proper nutrition; and which might be controlled or removed by a course of mercury, as constitutional syphilis and malaria often were; at any rate, it was possible that it might produce a new action in his system. This he dreaded very much, and offered a great many objections, which I removed; but he declined it for the present.

June 17th. To-day Mr. R. consented to take mercury. I gave him a one gr. blue pill, foui times a day, with an occasional aperient, and continued the opium to relieve his suffering. I applied blisters over various parts of his spine, which increased his pain so much, that I was obliged to heal them directly. This treatment was continued about three weeks, with an occasional rubbing in of mercurial ointment over his abdomen, when a considerable improvement was manifest. Treatment continued.

July 15. Was summoned to Mr. R., when I expected another unfavorable turn in his disease had taken place, but was agreeably disappointed in finding him much relieved and improving, and down stairs. The statement which I had before repeatedly made to him, that he presented all the phenomena of poisoning with lead, made a very strong impression on his mind, so much so, that it constantly occupied his mind, and just brought to his recollection that he had been in the habit for many years of chewing lead, and that this habit extended so far back, that he was unable to date its commencement. Formerly, being very fond of his gun, he frequently took hunting excursions, on which occasion he always had a piece of bullet or shot in his mouth; when in the store, he seldom ever passed by the box containing the shot, without putting some in his mouth to chew. But, what he most liked from its agreeable taste, and of which he chewed a great deal, was the lead lining of tea boxes; besides, he considered that the presure of the teeth on the metal enabled him better to bear his pain. I immediately replied, that the cause of all his suffering and bad health was perfectly clear, and at once assured him that he might yet be a sound man. I at once examined his gums for Dr. Burton's symptom, and found the blue line over four or five teeth. I considered the case fairly made out, and never felt so much rejoiced as at that moment, to think, that an individual, after such a prolonged period of suffering and bad health, whom all considered as beyond recovery, and almost in the grave, should by this discovery be yet restored to health and usefulness. Not so my patient, however; he was very skeptical of my prognosis, not conceiving it possible that his disease could have originated from what to him appeared so slight a cause. I assured him that his case always appeared a very strange one to me, and I was always astonished to think that a healthy individual as he had always been, should have been reduced to such a protracted state of bad health, without any organic disease, unless from some evident cause, which had at last been discovered; that it was now rendered almost certain, that his first attack, which was considered acute peritonitis—and many subsequent ones, were attacks of lead colic. Further, that his attack of autumnal fever, from which I date the commencement of his disease, had probably produced a debilitated state of the body, rendering it more susceptible to the influence of minute portions of the metal; also, that nearly every symptom, which writers have laid down as indicating poisoning with lead, had in his case been repeatedly and severely manifested; and the only reason why he did not before recover, was the continued renewal of the poison, whenever he was present where it could be obtained. Also, that we were now in a fair way of proving it, the cause being discovered, would in future be avoided, and he would continue well. I pointed to the present amelioration of his disease from the treatment he was pursuing, as a favorable indication that it depended on some removable cause, as idiopathic neuralgia of such long standing was seldom benefitted by any treatment. From all this, it will be seen that I had to urge a number of reasons to convince my patient of the real nature of his case, but without convincing him. I added acid sulph. aromat. to the former remedies, and by the 1st of August, his pains had nearly entirely subsided, his bowels were acting naturally, and he left off medicine, even opium, for the first time since the commencement of his sickness. I ordered splints to his wrists and hands, which gradually recovered their natural state.

Mr. R. entirely recovered his health in every respect, and has continued well up to the present time, February, 1850, being again a *strong, active* business man. *He thinks* that the extensor muscles of his wrists and fingers are not *quite* so strong as before his disease commenced, which is probably true, as muscles which have long been inactive, require frequent and strong exercise to recover their proper tone, which cannot be given to these muscles; their function being merely extension, they cannot be exercised to any extent; however, the defect is very slight indeed.— *Western Lancet.*

